# Molecular uncovering of important helminth species in wild ruminants in the Czech Republic

**DOI:** 10.3389/fvets.2025.1544270

**Published:** 2025-02-04

**Authors:** Lucie Škorpíková, Jaroslav Vadlejch, Jana Ilgová, Radim Plhal, Jakub Drimaj, Ondřej Mikulka, Jan Magdálek, Martin Kašný, Nikol Reslová

**Affiliations:** ^1^Department of Botany and Zoology, Faculty of Science, Masaryk University, Brno, Czechia; ^2^Department of Zoology and Fisheries, Faculty of Agrobiology, Food and Natural Resources, Czech University of Life Sciences Prague, Prague, Czechia; ^3^Department of Forest Protection and Wildlife Management, Faculty of Forestry and Wood Technology, Mendel University in Brno, Brno, Czechia

**Keywords:** *Ashworthius sidemi*, *Fascioloides magna*, *Haemonchus* spp., rumen flukes, wild ruminants, multiplex real-time PCR, nested PCR, environmental fecal samples

## Abstract

Monitoring gastrointestinal helminth infections in wild ruminants poses significant challenges for managing wildlife health, particularly regarding invasive species. Traditional coprological methods are often limited by their labor-intensive nature and potential for erroneous identification due to morphological similarities among parasite species. This study employed advanced molecular techniques to assess the prevalence and distribution of several helminth taxa, including the invasive nematode *Ashworthius sidemi* and the trematode *Fascioloides magna*, in wild ruminant populations in the Czech Republic (CR). A comprehensive and extensive survey on parasite occurrence, unique in its nationwide scope, was conducted on 983 fecal samples collected from red deer (*Cervus elaphus*), roe deer (*Capreolus capreolus*), fallow deer (*Dama dama*), and mouflon (*Ovis musimon*) across various regions of the CR. The samples were analyzed using multiplex real-time PCR assays specifically designed to detect the DNA of six helminth representatives: the nematodes *A. sidemi* and *Haemonchus* spp., as well as the trematodes *F. magna*, *Dicrocoelium dendriticum*, *Fasciola hepatica*, and *Calicophoron daubneyi* (and representatives of the family Paramphistomidae, respectively). These assays targeted regions of ribosomal DNA (rDNA) and were designed to exhibit high sensitivity and specificity, enabling accurate detection of helminth parasites directly in fecal samples. The molecular assays revealed that invasive nematode *A. sidemi* was the most prevalent helminth species, detected in 15.8% of all samples (155/983), with the highest infection rate observed in red deer at 30.7% (124/404). *Haemonchus* spp. were also frequently detected, identified in 14.9% of samples (146/983), particularly in roe deer, with a prevalence of 23.2% (86/371). Spatial analysis of these nematodes across various regions of the CR revealed the extensive distribution of both *A. sidemi* and *Haemonchus* spp. in nearly all regions. In contrast, trematode infections were less common, with *F. magna* and *D. dendriticum* each found in only 1.5% of samples (15/983). Members of the family Paramphistomidae were detected in 0.2% of the samples (2/983) and were confirmed through sequencing as *C. daubneyi*. The geographical distribution patterns identified in this study indicate potential hotspots for specific helminth species. These findings are critical for planning health management and conservation strategies to mitigate the impacts of helminth infections, especially in areas affected by invasive species.

## 1 Introduction

In recent decades, globalization, climate change, and human activities have significantly transformed biological communities, resulting in increased introductions of non-native flora and fauna into new habitats, either intentionally or accidentally (1–3). Non-native species that successfully adapt to new environments and establish self-sustaining populations are considered invasive species. These species can outcompete native organisms, disrupt ecosystems, and may have profound consequences for biodiversity and even the health of native biota (4–6). A detailed understanding of invasive species and their ecological impacts is crucial for developing effective management strategies (7–9).

A critical concern accompanying the introduction of non-native species is the inadvertent co-introduction of their associated pathogens, termed “biological hitchhikers” (10–12). In parasitology, these “hitchhikers” can exploit the movement of the native host species to spread to new areas. This may disrupt local host–parasite dynamics, endanger native species, and alter population structures, thereby leading to long-term ecological consequences. A striking illustration of such “biological hitchhikers” can be seen in the co-introduction of gastrointestinal parasites such as *Ashworthius sidemi* and *Fascioloides magna* (13–15). These species illustrate how human activities, such as the translocation of deer species across continents, have facilitated the geographic dispersal of these parasites, which are now considered invasive.

The strongylid nematode *A. sidemi* was co-introduced into the CR alongside the sika deer (*Cervus nippon*) during the late 19th and early 20th centuries (16). Originally native to East Asia, sika deer were imported into several European countries to enrich local game stocks during this period (17). Many of these deer, originally confined to game reserves, escaped or were released into the wild and have since thrived in the European climate, resulting in a notable population increase. In this context, *A. sidemi* has successfully adapted to the European climatic and environmental conditions and has begun infecting indigenous ruminants in various European countries (18–21). Presently, it is found in nearly all wild ruminant species inhabiting Europe.

The introduction of the giant liver fluke, *F. magna*, into the CR followed a similar pattern. The human-mediated translocation of white-tailed deer (*Odocoileus virginianus*) or wapiti deer (*Cervus canadensis*) since the latter half of the 19th century facilitated the geographical expansion of *F. magna* from its native range in North America to Europe (22–24). This liver fluke represents a typical example of a parasite that has successfully established invasive populations, adapting to indigenous European ruminants as definitive hosts and using local snail species as intermediate hosts (25–28).

Moreover, *Calicophoron daubneyi*, formerly classified as *Paramphistomum daubneyi*, has emerged as another potentially significant candidate with invasive capabilities. This is because members of the family Paramphistomatidae were historically seldom documented in the temperate climate of Europe (29–31). However, subsequent reports have identified an increasing prevalence of rumen flukes and their negative impact on livestock farming. Now, molecularly confirmed *C. daubneyi* has become the common species in farm ruminants across various European countries (31–33), including the CR (30, 34). Records of its presence in the wild further highlight its extensive distribution (29, 35, 36).

In addition to helminth species with recognized invasive potential, this study also examines non-native and native yet ecologically important parasitic taxa, specifically nematodes of the genus *Haemonchus* and liver flukes *Fasciola hepatica* and *Dicrocoelium dendriticum*.

The genus *Haemonchus*, with *Haemonchus contortus* as its most notable representative, comprises parasitic nematodes with a global distribution and a broad host range (37–39). These blood-feeding parasites predominantly inhabit the abomasum of ruminants, causing severe health issues such as profound anemia and reduced fitness, particularly in small domestic ruminants. While their impact on domestic livestock is well-documented, *Haemonchus* spp. also demonstrates the capacity to infect wild ruminants, for example, roe deer (*Capreolus capreolus*), red deer (*Cervus elaphus*), or fallow deer (*Dama dama*). This includes populations across diverse ecosystems, such as those in the CR (18, 40). The interactions between wild and domestic ruminants are of particular concern, as wild ruminants may act as reservoirs for *Haemonchus* spp., including strains resistant to anthelmintic treatments (37, 41, 42).

*Fasciola hepatica*, commonly known as the liver fluke, is a globally distributed parasitic trematode predominantly affecting grazing animals, including both domestic and wild ruminants (43–45). Its complex life cycle necessitates definitive hosts, such as ruminants, and intermediate hosts, primarily freshwater snails from the Lymnaeidae family. Similar to *Haemonchus* spp., *F. hepatica* has been extensively investigated in domestic livestock due to its profound economic and veterinary significance. However, its epidemiology and ecological dynamics within wild ruminant populations remain underexplored and warrant further scientific attention.

The last parasite of interest, *D. dendriticum*, commonly known as the lancet fluke, is a globally distributed trematode that primarily parasitizes the bile ducts of domestic and wild ruminants (46, 47). This parasite exhibits a complex life cycle involving terrestrial snails and ants as intermediate hosts, enabling its adaptation to diverse ecosystems, particularly in dry, mountainous, and lowland pastures. Wild ruminants, including red deer (*C. elaphus*), roe deer (*C. capreolus*), and mouflons (*Ovis musimon*), are significant definitive hosts in Europe, facilitating the persistence and transmission of *D. dendriticum* within natural habitats (48–50). However, comprehensive knowledge regarding the prevalence of this parasite in wild ruminant populations remains limited.

To effectively study parasites, whether invasive or native, reliable detection techniques and accurate taxonomic identification are critical (12, 51, 52). These tools are essential for monitoring their distribution, understanding genetic diversity, and studying population dynamics. Managing the spread of invasive parasites is crucial due to their potential to disrupt local ecosystems, impact biodiversity, and cause significant economic and health-related consequences. Traditional coprological methods, such as flotation or sedimentation, are conventionally used to recover parasite developmental stages from host feces, relying on distinct morphological characteristics for identification (53–55). Despite their widespread use, these methods have limitations, including the potential for morphological similarities between species, leading to incorrect taxonomic classification (56–58). To overcome these challenges and enhance accuracy in species identification, applying highly sensitive and specific molecular approaches is often necessary.

Therefore, the aim of this study, representing a unique survey at the national level, is to comprehensively assess the current spread of invasive helminth species, including *A. sidemi*, *F. magna*, and *C. daubneyi*, supplemented by other important representatives such as *Haemonchus* spp., *F. hepatica*, and *D. dendriticum*, in the most common wild ruminant species in the CR. To ensure the accurate taxonomical determination of parasite species, advanced molecular techniques employing real-time PCR with TaqMan probes were employed to detect parasite DNA in animal feces directly. These molecular approaches are universally applicable to invasive, non-invasive, and native helminth species, enabling comprehensive monitoring and precise identification. They also provide a non-invasive means to assess the prevalence and distribution of helminths, allowing for prevalence quantification as the proportion of positive samples relative to the total analyzed. By conducting a large-scale survey on wild ruminant populations across diverse regions in the country, this research provides insights essential for managing and effectively mitigating the impacts of these invasive parasites on local ecosystems and animal health.

## 2 Materials and methods

### 2.1 Geographic and climatic context

The study was conducted in the CR, which is characterized by a temperate continental climate marked by pronounced seasonal variations, including warm summers and cold winters. According to data from the Czech Hydrometeorological Institute (CHMI), the average annual temperature during the specific sampling period from March 2022 to April 2024 was 9.5°C. Within this timeframe, January represented the coldest month, with an average temperature of 0.9°C, while July was the warmest month, averaging approximately 19.1°C. Precipitation was evenly distributed throughout the year, with total annual precipitation during this study period amounting to 731.5 mm, peaking in the summer months, particularly in July and August. The country features diverse topography, encompassing mountainous regions, lowland areas, and significant river basins, notably those of the Vltava and Elbe rivers. To maintain conceptual clarity, we considered the country’s administrative division into 14 regions (the capital city of Prague, Central Bohemia, South Bohemia, Plzeň, Karlovy Vary, Ústí nad Labem, Liberec, Hradec Králové, Pardubice, Vysočina, South Moravia, Zlín, Olomouc, and Moravian-Silesian regions), though the primary focus remained on national-level findings.

### 2.2 Fecal sample collection

**Figure 1:**
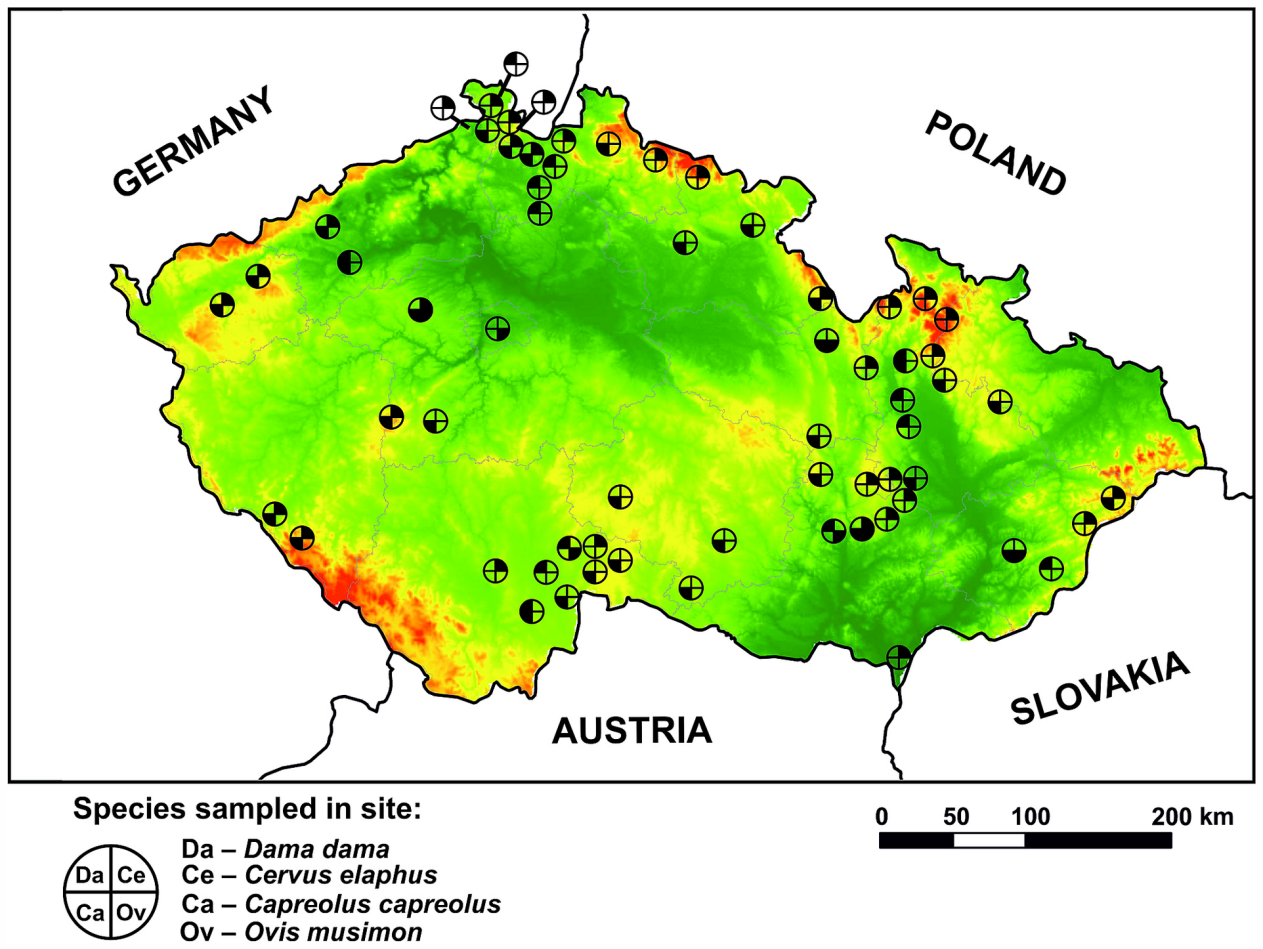
Spatial distribution of sampling sites for wild ruminant species (*Dama dama*, *Cervus elaphus*, *Capreolus capreolus*, and *Ovis musimon*) within the Czech Republic. Lowlands are presented in green color while highlands are in red color.

Between March 2022 and April 2024, voided fecal pellets were gathered from various aggregating spots of wild ruminants, such as feeding sites, migration paths, holding covers, and game reserves across different regions of the CR. The final sample set consisted of 371 samples of roe deer (*Capreolus capreolus*) from 33 locations, 404 samples of red deer (*Cervus elaphus*) from 32 locations, 130 samples of fallow deer (*Dama dama*) from 13 locations, and 78 samples of mouflon (*Ovis musimon*) from 7 locations (for more information, please see Supplementary material 1). Red deer and roe deer were prioritized due to their status as native ruminant species in the CR. The locations where the collection occurred are marked on the map (Figure 1). The collection predominantly occurred during cold seasons to ensure the freshness of the feces and minimize their coverage by growing vegetation. Both temporal and spatial stratification of fecal piles was carefully observed to maximize sampling from individual animals. Approximately 5 g of feces per sample was collected and stored at −20°C in plastic zip-lock bags for subsequent molecular analyses.

### 2.3 DNA extraction from fecal samples

Total DNA extraction followed the procedure outlined in (58). Initially, frozen fecal samples were thawed and manually shredded in zip-lock bags. Subsequently, 1 g of feces was used for DNA extraction using the Quick-DNA Fecal/Soil Microbe MiniPrep kit (Zymo Research, Irvine, CA, USA). To ensure thorough grinding of the fecal pellets, 800 μL of BashingBead Buffer and 3,200 μL of PBS were added to each sample and mixed thoroughly. A total of 1,200 μL of this suspension was transferred into a ZR BashingBead Lysis Tube and mechanically homogenized for 10 min using a Retsch MM200 mixer mill (RETSCH, Haan, Germany) with a vibrational frequency of 1,800 rpm. Additionally, for every set of about 20 samples (representing samples from two locations), a negative isolation control (NIC) containing only buffers without fecal matter was included in the extraction process. The following procedure was carried out in accordance with the manufacturer’s protocol provided in the kit. Subsequently, 50 μL of total DNA eluate of each sample was stored at −20°C for future analyses.

### 2.4 DNA extraction from reference helminth specimens

Mature helminths recovered from the gastrointestinal tracts of deceased ruminants during standard parasitological post-mortem examinations were taxonomically classified based on distinctive morphological characteristics viewed under an Olympus BX51 light microscope with specialized measuring software QuickPHOTO MICRO 3.0 software (PROMICRA, Prague, CR). Subsequently, genomic DNA was extracted from individual helminths of each species using commercially available kits (e.g., DNeasy Blood & Tissue Kits, Qiagen, Germany) or other established DNA extraction protocols (59). The isolated gDNA were assessed for concentration and purity using a Qubit fluorometer (Thermo Fisher Scientific, Waltham, MA, USA) and stored at −20°C. If necessary, PCR tests employing varied combinations of universal primers were conducted to ensure accurate species or genus assignment as detailed in (60–67). The resulting purified amplicons underwent Sanger sequencing, and the obtained sequences were compared *in silico* with data available in the NCBI GenBank sequence database.^1^

### 2.5 Design and optimization of detection systems for trematodes

Assays development involved designing primers and dual-labeled hydrolysis probes with a reporter dye (FAM, HEX, or ROX) at the 5′ ends and a compatible dark quencher at the 3′ ends. To achieve this, candidate gene sequences of the selected trematode species were downloaded from the NCBI GenBank sequence database and aligned to identify conserved species-specific regions optimal for oligonucleotide hybridization. The selected sequences were evaluated using the online tool OligoAnalyzer 3.1^2^ to ensure appropriate parameters for assay performance. The specificity of each primers-probe system was assessed using nucleotide BLAST,^3^ enabling its comparison with all publicly available nucleic acid sequences.

### 2.6 Nested real-time PCR assay for detection of *F. hepatica* and rumen flukes

The first round of amplification of DNA, which was isolated from fecal samples, was performed using standard PCR. The amplification of the target region employed a universal pair of primers for flukes: GA1 and BD2, as referenced by Luton et al. (66) and Anderson and Barker (67). The reaction was conducted in a total volume of 20 μL, containing PPP Master Mix (Top-Bio, Prague, CR), 0.125 μM of each primer, ultrapure PCR-grade H_2_O, and 5 μL of total DNA. The amplification protocol included an initial denaturation step at 95°C for 1 min, followed by 20 cycles of 95°C for 30 s, 55°C for 30 s, and 72°C for 45 s, with a final elongation step at 72°C for 5 min. The resulting PCR products were subsequently diluted sixfold with ultrapure PCR-grade H_2_O.

The second round of amplification involved a real-time triplex PCR assay designed to simultaneously detect a specific region of *C. daubneyi* and *F. hepatica*. Additionally, an extra primer was incorporated into the assay to non-specifically detect other representatives of the family Paramphistomidae, with a particular focus on *P. cervi* or *P. leydeni*. The third primers-probe system was added to detect the sequence for internal amplification control (IAC), as described below.

The reaction mixture, in a final volume of 20 μL, consisted of 1X Luna Universal Probe qPCR Master Mix (New England Biolabs, Ipswich, MA, USA), 250 nM of each of the seven primers, 100 nM of FAM probe, 100 nM of Cy5 probe, 100 nM of ROX probe, ultrapure PCR-grade H_2_O, 1 × 10^5^ copies of IAC plasmid, 0.4 U UDG, and 5 μL of diluted products from the first round of PCR. All reactions were conducted under the following conditions: carryover prevention at 37°C for 10 min and 95°C for 10 min, initial denaturation at 95°C for 2 min followed by 40 cycles of denaturation at 95°C for 15 s, and annealing/extension at 60°C for 45 s. A final cooling cycle at 40°C for 30 s was then included.

For a comprehensive overview of the sequences of probes and primers used in the various detection systems, including amplicon size and specific target details, please refer to Supplementary Table 1.

### 2.7 Nested real-time PCR assay for detection of *F. magna* and *D. dendriticum*

To address contamination issues during product transfer in nested PCR, a one-step, single-tube nested real-time PCR assay for the detection of *F. magna* was developed, building on the work of Wang et al. (68). External primers were designed to target a sequence of the repetitive sequence region *5.8S*-*ITS2*-*28S*, resulting in a 523 bp amplicon. With annealing temperatures distinct from these external primers, internal primers were subsequently used to amplify segments of the *ITS2* region specifically detected by a probe. Additionally, a primers-probe system targeting the *ITS1* region of *D. dendriticum* was integrated into the assay to identify potential false-positive results that may arise during the detection of *F. magna* and *F. hepatica* (see the subsection on specificity and sensitivity for further details). Furthermore, a third primers-probe system was incorporated to detect the sequence for the IAC, as described below.

The reaction mixture, with a final volume of 20 μL, comprised 1X Luna Universal Probe qPCR Master Mix (New England Biolabs, Ipswich, MA, USA), 75 nM of each of the external primers, 250 nM of each of the remaining six primers, 100 nM of the FAM probe, 100 nM of the Cy5 probe, 100 nM of the HEX probe, ultrapure PCR-grade H_2_O, 1 × 10^5^ copies of IAC plasmid, 0.4 U UDG, and 5 μL of total DNA. All reactions were carried out under the following conditions: carryover prevention at 37°C for 10 min and 95°C for 10 min, initial denaturation at 95°C for 2 min, followed by 15 cycles of denaturation at 95°C for 15 s, and annealing/extension at 70°C for 45 s. This was followed by 40 cycles of denaturation at 95°C for 15 s and annealing/extension at 62°C for 45 s. A final cooling step at 40°C for 30 s was then included.

For a comprehensive overview of the sequences of probes and primers used in the various detection systems, including amplicon size and specific target details, please refer to Supplementary Table 2.

### 2.8 Real-time PCR assay for detection of *A. sidemi* and *Haemonchus* spp.

The primers and probes were used to identify two representatives of blood-sucking gastrointestinal nematodes, *A. sidemi* and *Haemonchus* spp. (encompassing species *H. contortus* and *H. placei*). The composition of the reaction mixture and cycling parameters were adopted from the publication (58). Therefore, real-time triplex PCR was implemented to simultaneously detect specific regions of internal transcribed spacers (*ITS*), specifically *ITS1* for *A. sidemi* and *ITS2* for *Haemonchus* spp. The third target was designed to detect the sequence for IAC, as described below.

The reaction mixture, in a final volume of 20 μL, consisted of 1X Luna Universal Probe qPCR Master Mix (New England Biolabs, Ipswich, MA, USA), 250 nM of each of the six primers, 100 nM of FAM probe, 100 nM of Cy5 probe, 200 nM of HEX probe, ultrapure PCR-grade H_2_O, 1 × 10^5^ copies of IAC plasmid, 0.4 U of Antarctic thermolabile UDG (New England Biolabs, Ipswich, MA, USA), and 5 μL of template DNA. All reactions were performed under the following conditions: carryover prevention at 37°C for 10 min, initial denaturation at 95°C for 2 min, followed by 40 cycles of denaturation at 95°C for 15 s, and annealing/extension at 57°C for 45 s. Finally, one cooling cycle at 40°C for 30 s was included.

For a comprehensive overview of the sequences of probes and primers used in the various detection systems, including amplicon size and specific target details, please refer to Supplementary Table 3.

### 2.9 Specificity and sensitivity determination

Trematode systems that exhibited appropriate parameters and passed the initial *in silico* specificity screening, as well as the assay developed for the detection of *A. sidemi* and *Haemonchus* spp., were experimentally tested using equimolar amounts of template gDNA (2 ng) extracted from mature helminths found in wild or domestic ruminants during post-mortem examinations. These included the representatives of the following trematodes: *F. hepatica*, *F. magna*, *D. dendriticum*, *C. daubneyi*, *P. cervi*, *Parafasciolopsis fasciolaemorpha*, and nematodes: *H. contortus*, *A. sidemi*, *Capillaria* sp., *Chabertia ovina*, *Oesophagostomum venulosum*, *O. columbianum*, *Bunostomum* spp., *Nematodirus filicollis*, *Ostertagia leptospicularis*, *Trichostrongylus vitrinus*, *T. axei*, *T. capricola*, *T. colubriformis*, *Teladorsagia circumcincta*, *Trichuris ovis*, *T. discolor*, *Cooperia* spp., and *Spiculopteragia* spp.

Although the in-silico screening did not identify any potential off-target matches, the detection systems for *F. magna* and *F. hepatica* exhibited cross-reactivity with the DNA of the trematode *D. dendriticum* at concentrations up to 200 pg per reaction, necessitating the inclusion of this target in the assay. However, based on the analysis of real samples, no such cross-reactivity was observed in practice. Furthermore, no non-specific reactions with other tested parasite species were observed in the detection systems for *C. daubneyi*, *D. dendriticum*, *Haemonchus* spp., and *A. sidemi*, while the signal for the IAC was positive in all reactions. Incorporating an additional primer into the *C. daubneyi* system demonstrated the capability to detect various representatives of the family Paramphistomidae (evidenced by testing on the gDNA of *P. cervi* and *C. daubneyi*).

The sensitivity of each assay was initially evaluated in individual reactions for each system and subsequently validated in a multiplex format. A DNA concentration gradient ranging from 1 ng to 100 ag per reaction was utilized for these tests. For mixed infection assessments, the gDNA of both target helminths was combined and diluted similarly. Furthermore, mixed infections were analyzed, with one target present in excess and another in trace amounts. To assess whether non-competitive environmental DNA could interfere with the assays, 10 μg of excess DNA from fish sperm (SERVA, Heidelberg, Germany) was added to the samples before testing. The detection limits established for the real-time PCR assays were as follows: 1 pg for *F. hepatica*, 1 pg for *P. cervi*, 100 fg for *C. daubneyi*, 10 fg for *F. magna*, 100 fg for *D. dendriticum*, 1 pg for *A. sidemi*, and 100 fg for *H. contortus*.

### 2.10 Validity of assays and data evaluation

All samples were analyzed in duplicate using a LightCycler 480 Instrument II (Roche, Basel, Switzerland) with 96-well PCR plates. A no-template control (NTC; i.e., 5 μL of ultrapure PCR-grade H_2_O was added instead of isolated DNA), along with the NICs and positive controls, were included on each analyzed plate. Additionally, the IAC was incorporated into every reaction, including all other controls. This non-competitive sequence was designed from ancient mitochondrial DNA sequences of two extinct species (*Thylacinus cynocephalus*, Sequence ID: FJ515781.1, and *Dinornis struthoides*, Sequence ID: AY326187.1) as described by Mikel et al. (69). The IAC sequence was synthesized *de novo* (Sigma-Aldrich, St. Louis, MO, USA), cloned into a plasmid, purified, and diluted using the same procedure described by Reslova et al. (58). Consequently, the reaction premixes of all multiplex assays included IAC-specific primers, a hydrolysis probe, and the plasmid construct containing the IAC sequence, functioning as a template to differentiate between truly negative and false-negative reactions where amplification inhibition occurred.

The results were analyzed using the FAM, HEX, or ROX channels in LightCycler software (version LCS480 1.5.1.62) with the “Fit Points” analysis method. When using a combination of FAM and HEX, universal fluorescent color compensation for both channels was enabled using the Universal CC FAM (510) – VIC (580) [465–510, 533–580] settings. For the Cy5 channel, which detects the IAC, the “2nd Derivative Max” analysis method was employed. If all controls performed as expected, a sample was considered positive only when both replicates yielded positive signals. The analysis and/or DNA extraction were repeated if these conditions were unmet.

### 2.11 Statistical analyses

Chi-square tests were performed to assess the significance of differences in the prevalence of *A. sidemi* and *Haemonchus* spp. among the tested ruminant species. The relationship between host population density and parasite infection prevalence was evaluated using Pearson correlation analysis. Helminth infection prevalence was correlated with the population densities of ruminant species in each location, estimated from hunting data as the number of animals culled per 1,000 hectares. Additionally, we analyzed the prevalence of single infections and co-infections within our dataset (*n* = 983), categorizing the samples into four groups: no infection (*n* = 687), infection with *A. sidemi* only (*n* = 150), infection with *Haemonchus* spp. only (*n* = 141), and co-infection with both parasites (*n* = 5). The probability for each category was calculated as the proportion of samples in that category relative to the total sample size. The expected probability of co-infection, assuming the independent occurrence of the two parasites, was calculated as the product of their individual probabilities. A Chi-square test was then used to compare the observed frequency of co-infections with the expected frequency. All statistical analyses were conducted at a 0.05 significance level.

## 3 Results

The overall prevalence of each examined helminth is summarised in Table 1. The data indicate that nematodes of the genus *Haemonchus* and the invasive species *A. sidemi* exhibit comparable prevalence rates of 14.9 and 15.9%, with 146 and 155 occurrences, respectively. In contrast, liver and rumen flukes were infrequently detected, with *F. magna* and *D. dendriticum* present only in 15 samples (*p* = 1.5%). Notably, *F. hepatica* was absent in all samples, while members of the family Paramphistomidae were detected in just two cases (*p* = 0.2%). Both were identified as *C. daubneyi* based on sequencing analysis, showing 100% sequence similarity with GenBank Accession No. MN044947.1.

**Table 1:** The overall prevalence of target helminth representatives among various species of wild ruminants occurring in the Czech Republic.

		Helminth species					
		*Ashworthius sidemi*	*Haemonchus* spp.	*Fascioloides magna*	*Fasciola hepatica*	*Dicrocoelium dendriticum*	Paramphistomidae
Ruminant species	Red deer (*C. elaphus*)	30.7% (124/404)	6.9% (28/404)	3.0% (12/404)	0.0% (0/404)	0.0% (0/404)	0.0% (0/404)
	Roe deer (*C. capreolus*)	5.4% (20/371)	23.2% (86/371)	0.5% (2/371)	0.0% (0/371)	0.8% (3/371)	0.0% (0/371)
	Fallow deer (*D. dama*)	7.7% (10/130)	16.9% (22/130)	0.8% (1/130)	0.0% (0/130)	5.4% (7/130)	0.8% (1/130)
	Mouflon (*O. musimon*)	1.3% (1/78)	12.8% (10/78)	0.0% (0/78)	0.0% (0/78)	6.4% (5/78)	1.3% (1/78)
Total prevalence		15.8% (155/983)	14.9% (146/983)	1.5% (15/983)	0.0% (0/983)	1.5% (15/983)	0.2% (2/983)

Prevalence is expressed as a percentage, with the number of positive cases/total samples provided in parentheses.

Among the ruminant species, the highest proportion of positive findings was observed in red deer (38.9%), while the lowest was found in mouflon (19.2%). Fallow deer and roe deer exhibited intermediate rates of 27.7 and 28.0%, respectively. These findings indicated that red deer was the most heavily impacted host species by both nematodes and trematodes. When focusing exclusively on the prevalence of *A. sidemi* and *Haemonchus* spp. across the tested ruminant hosts, the analysis revealed statistically significant differences. The *p*-values for these differences were 1.212 × 10^−17^ for red deer, 9.154 × 10^−12^ for roe deer, 0.038 for fallow deer, and 0.012 for mouflon, indicating varying statistical significance across the host species. The most significant differences were noted in red deer and roe deer. These results indicate a possible host preference or increased susceptibility of red deer to *A. sidemi* (*p* = 30.7%), whereas *Haemonchus* spp. was most prevalent in roe deer (*p* = 23.2%). Moreover, Pearson correlation analysis indicated a positive relationship between *A. sidemi* prevalence in red deer and the estimated population density of this host species (*r* = 0.319), suggesting a notable increase in infection with rising red deer density. In contrast, the correlation for *Haemonchus* spp. in roe deer was moderate (*r* = 0.391), revealing a stronger association with higher population density and infection rates.

The results, summarised in Table 2, reveal several noteworthy concurrent infections involving multiple invasive parasite species. Specifically, we documented 10 cases of co-infection involving the species with invasive potential—the nematode *A. sidemi* and the trematode *F. magna*, and a single case of co-infection with *A. sidemi* and the trematode *C. daubneyi*. Additionally, an analysis focusing on the presence of representatives of the genus *Haemonchus* and the invasive species *A. sidemi* revealed the following probabilities: 69.9% without infection, 15.3% for infection with only *A. sidemi*, 14.3% for infection with only *Haemonchus* spp., and 0.5% for co-infection with these two nematodes. The expected probability of co-infection, calculated under the assumption of independent occurrences of the two parasites, was 2.2%. However, the observed co-infection probability of 0.5% was lower than the expected value. A Chi-square test conducted at the 0.05 significance level demonstrated a statistically significant difference (*χ*^2^ = 9.20, *p* = 0.0024), indicating that the lower frequency of co-infections is unlikely to be due to random chance. This finding indicates the possibility of a negative interaction, likely attributable to competitive exclusion, between *A. sidemi* and *Haemonchus* spp., which reduces the likelihood of simultaneous infection.

**Table 2:** Summary of co-infection findings observed across different host species.

		Helminth species			
		*Ashworthius sidemi* *Haemonchus* spp.	*Ashworthius sidemi* *Fascioloides magna*	*Ashworthius sidemi* *Calicophoron daubneyi*	*Haemonchus* spp. *Dicrocoelium dendriticum*
Ruminant species	Red deer (*C. elaphus*)	0	8 (same location)	0	0
	Roe deer (*C. capreolus*)	4 (different locations)	2 (different locations)	0	1
	Fallow deer (*D. dama*)	0	0	1	4 (same location)
	Mouflon (*O. musimon*)	1	0	0	1

**Figure 2:**
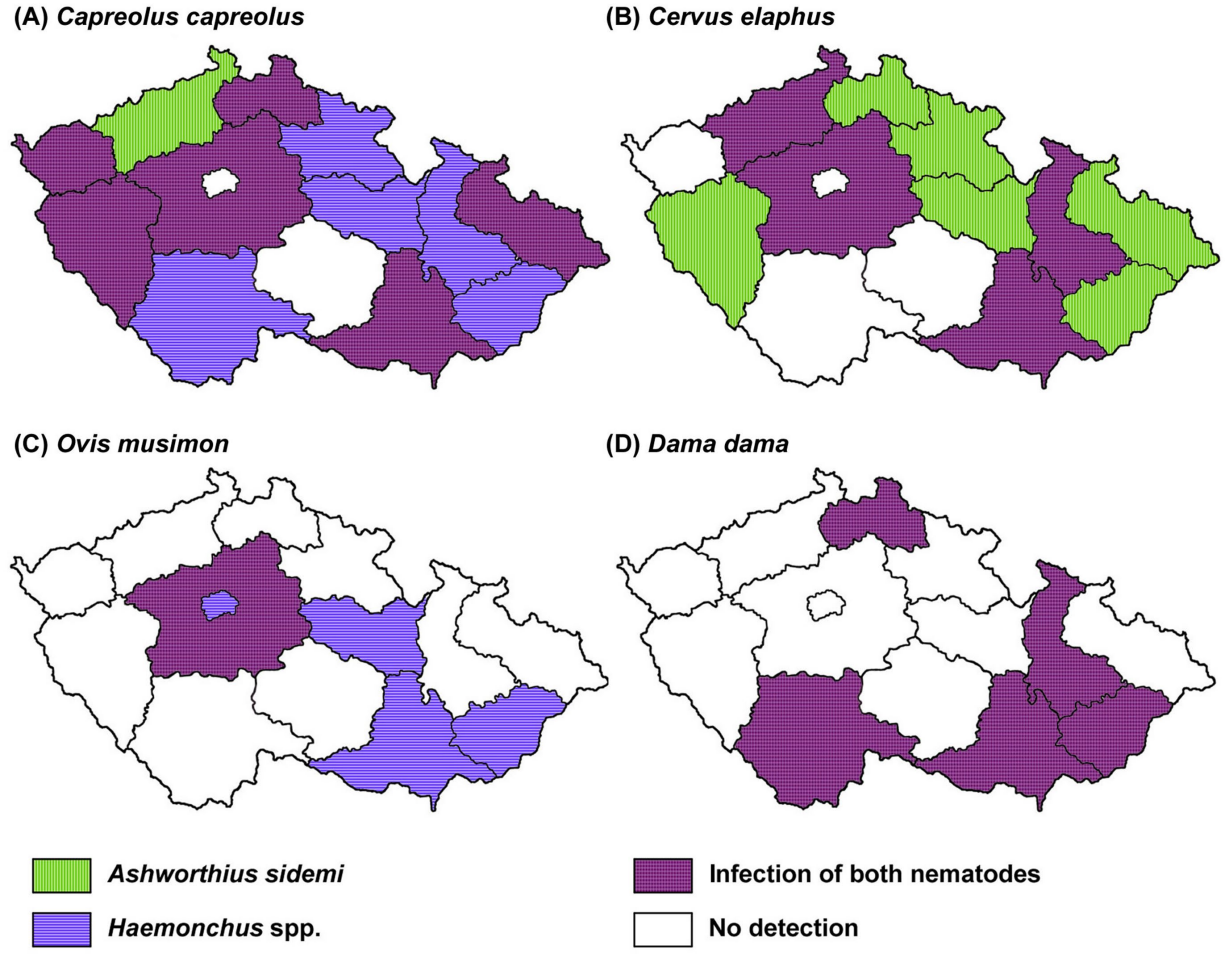
Geographical distribution of *Ashworthius* *sidemi* and *Haemonchus* spp. In various species of wild ruminants across different regions of the Czech Republic. Map showing the distribution of invasive parasites in **(A)** roe deer (*C. capreolus*), **(B)** red deer (*C. elaphus*), **(C)** mouflon (*O. musimon*) and **(D)** fallow deer (*D. dama*).

**Figure 3:**
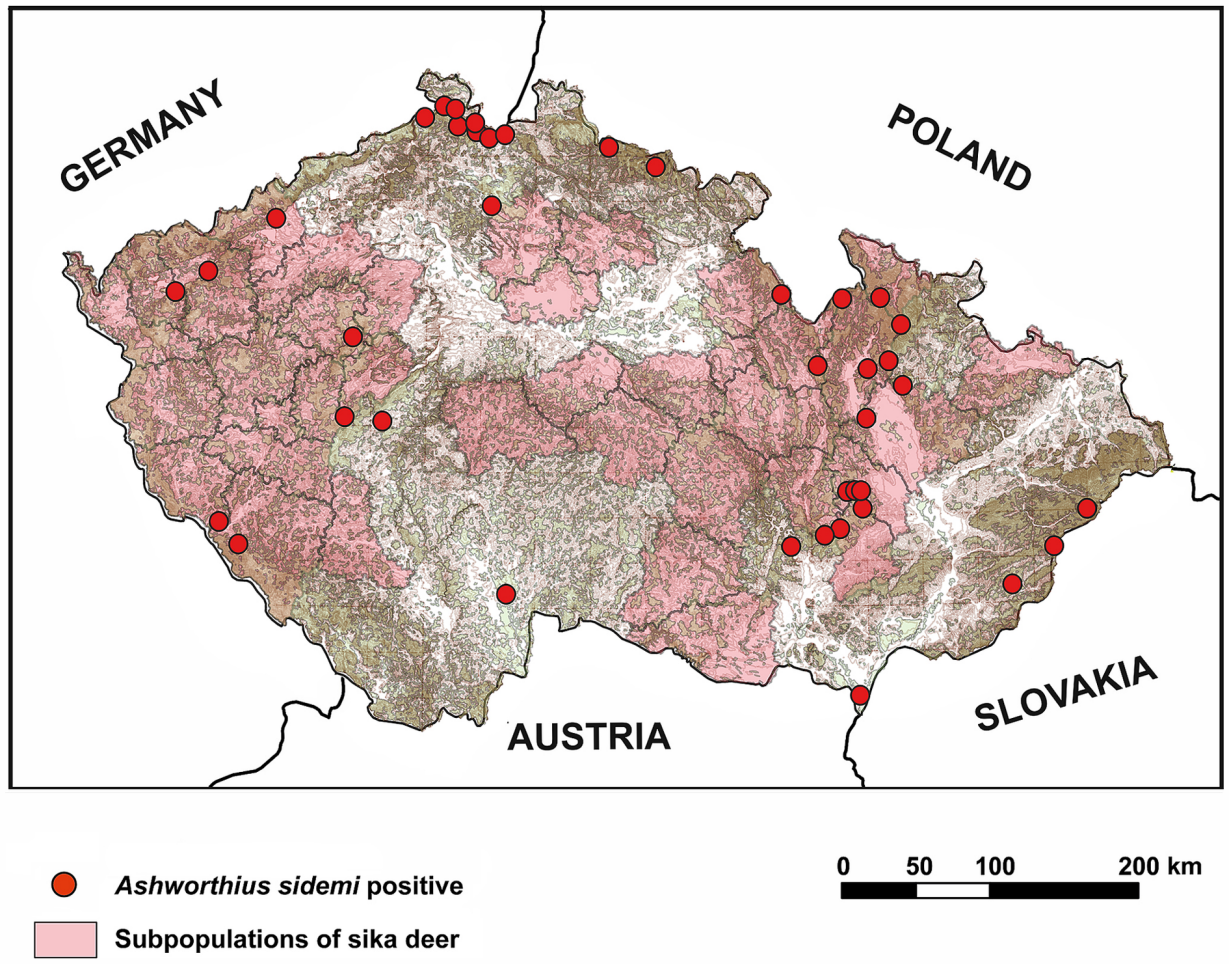
Geographical distribution of *Ashworthius sidemi* and its original host, sika deer (*Cervus nippon*), in the Czech Republic. The distribution of sika deer subpopulations was derived from data by Saggiomo et al. (73).

**Figure 4:**
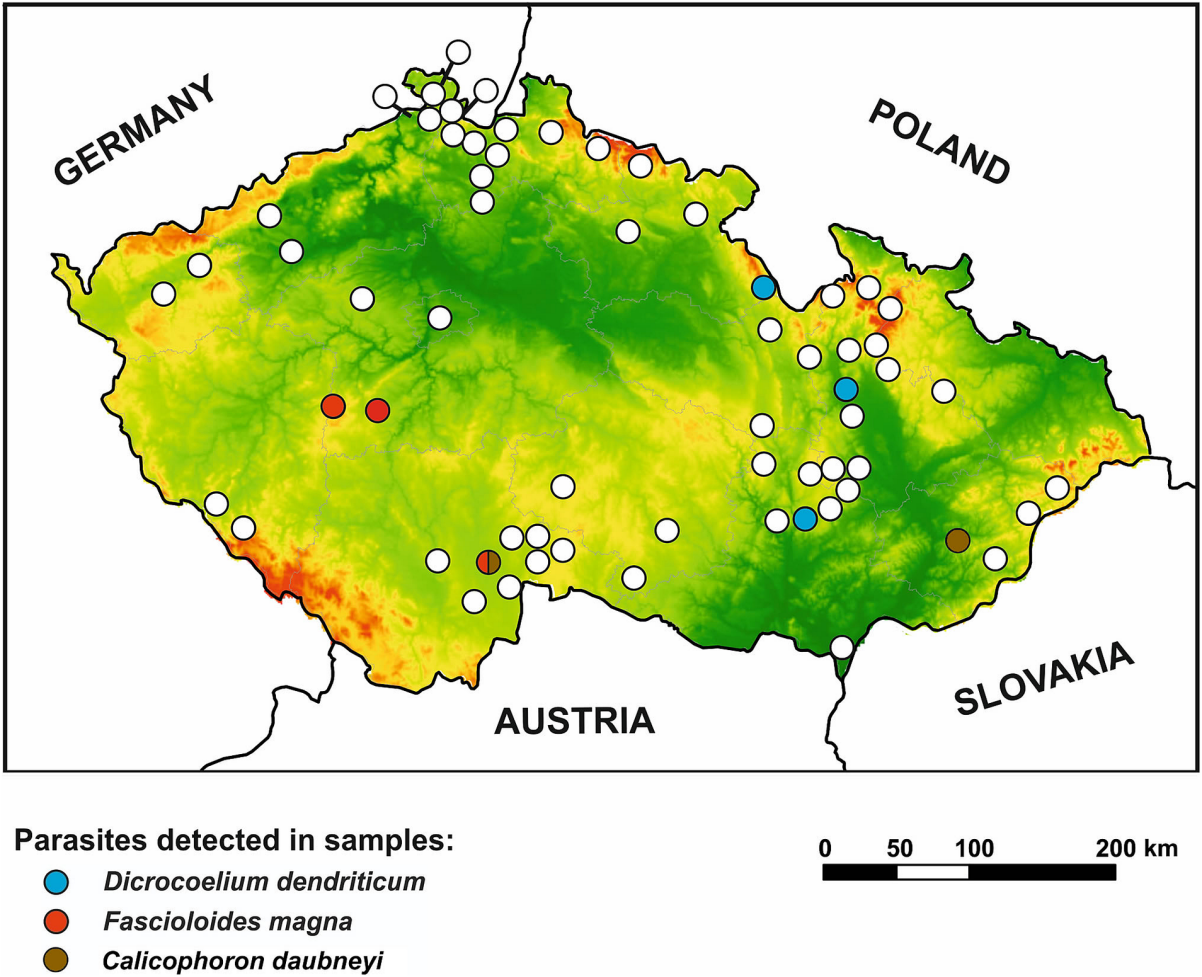
Geographical distribution of trematodes in wild ruminants in the Czech Republic. Positive locations for *Dicrocoelium dendriticum*, *Fascioloides magna*, and *Calicophoron daubneyi* are differentiated by color distinction. Lowlands are presented in green color while highlands are in red color.

A spatial analysis of parasite prevalence was conducted to identify potential geographical hotspots for parasite distribution. The data from our study indicate that the examined nematode species, *A. sidemi* and *Haemonchus* spp., were present across most regions of the CR, with no detection in the Vysočina region (and for *A. sidemi*, the capital city of Prague). The distribution patterns of both nematode species in relation to specific wild ruminant hosts are illustrated in Figure 2. Moreover, Figure 3 illustrates the distribution of *A. sidemi* alongside the range of sika deer populations across the CR, suggesting that this invasive parasite had expanded beyond the range of its original host. Regarding trematodes, in the Central Bohemian and South Bohemian regions, the invasive trematode *F. magna* was observed. Members of the family Paramphistomidae were identified in the South Bohemian, Zlín, and Hradec Králové regions. Additionally, *D. dendriticum* was detected in the South Moravian, Olomouc, and Hradec Králové regions. The locations where these trematodes were found are highlighted in Figure 4.

## 4 Discussion

The detection of an abomasal nematode *A. sidemi* in red deer, roe deer, and fallow deer in this study corroborates previous research demonstrating a pronounced host specificity of this parasite for cervids, likely a consequence of their co-evolutionary history (18, 21, 70). Furthermore, these findings align with earlier studies conducted in the CR, which have confirmed *A. sidemi* presence in these cervid species using both morphological and molecular methods (18, 71, 72). The widespread distribution of this parasitic nematode in cervid hosts throughout diverse regions of the CR provides strong evidence of its successful establishment and ongoing geographical expansion. This is supported by our data, as we detected *A. sidemi* in areas beyond the current range of sika deer, which, according to recent data (73), has not yet spread across the entire territory of the CR. Additionally, literature documenting the spread of *A. sidemi* in cervid populations across other European countries further supports its continued colonization of new habitats (19, 21, 74, 75).

Our results demonstrate a significant preference of *A. sidemi* for red deer or, alternatively, an increased susceptibility of this cervid host to this nematode. This is likely because red deer are phylogenetically more closely related to sika deer, the primary host for *A. sidemi*, than to other cervids such as roe deer. Additionally, a positive correlation was observed between the prevalence of *A. sidemi* in red deer and the population density of this host species. This underscores the pivotal role red deer populations play in maintaining this parasite and facilitating its transmission to further regions and other sympatric cervid species, including roe deer and fallow deer. According to our findings, *A. sidemi* was not detected in samples from the Vysočina region. This absence may be linked to the small red deer population in this region, the lowest observed across all regions, likely attributable to the restricted availability of suitable habitats (76).

Beyond the Cervidae family, *A. sidemi* has been identified in several species within the Bovidae family. Its presence has been documented in wild bovids such as the European bison (*Bison bonasus*) and chamois (*Rupicapra rupicapra*) (20, 77, 78). Concerning *A. sidemi* in mouflons (*O. musimon*), there is only a single mention of the potential transmission of this nematode species to this non-specific host, found in the study by Kotrla and Kotrly (79). The findings presented in our study contribute additional evidence supporting the occurrence of *A. sidemi* in this host species.

The widespread distribution and high prevalence rates of nematodes from the genus *Haemonchus* also warrant attention. These abomasal nematodes are well-established in wild ruminant populations in the CR. This is consistent with available data, which underscores the historical presence of this parasite in deer hosts in the CR over many years (18, 40, 72). Notably, patterns analogous to those observed between *A. sidemi* and red deer are also apparent in the strong affinity of *Haemonchus* spp. to roe deer. Similarly, a positive association was observed between the prevalence of these nematodes and the population density of roe deer. Our results corroborate the findings of a recent meta-analysis conducted by Brown and Morgan (37), which identified a significantly higher prevalence of *H. contortus* in roe deer compared to red deer and fallow deer. While their study reported a mean prevalence of 14.7% in roe deer across 11 out of 15 studies conducted in various countries across Europe, our data indicate an even higher prevalence with *Haemonchus* spp. detected in 23.2% of the roe deer sampled. This may be attributed to conditions unique to the CR, where roe deer are the most abundant species with high population densities sustained by landscape characteristics favorable to their habitat. This finding further suggests that roe deer function as a primary host species for *Haemonchus* spp., playing a crucial role as a reservoir for this nematode. Furthermore, our observations become more impactful given the pathogenic role of *Haemonchus* spp., with recent studies indicating that wild ruminants may serve as carriers of anthelmintic-resistant strains of this nematode (41, 42, 80).

Our results regarding the trematodes *F. magna* (*p* = 1.5%), *D. dendriticum* (*p* = 1.5%), as well as members of the family Paramphistomidae, specifically *C. daubneyi* (*p* = 0.2%), provide insight into their relatively restricted distribution compared to nematodes with direct life cycle. This limited prevalence aligns with their specific climatic and environmental requirements, including the need for intermediate hosts, such as snails, to complete their complex life cycles (81–83). The primary significance of these trematode species lies in their profound impact on the health and economic productivity of domestic ruminants, including sheep, goats, and cattle (22, 30, 84). Their pathogenic effects result in significant declines in productivity and critically impair overall animal health. Effective control of these parasites demands intricate and costly interventions, encompassing therapeutic treatments for infected animals and comprehensive preventive strategies to disrupt their life cycles. Therefore, our findings in wild ruminants highlight the need for targeted management efforts in regions where these trematodes have been confirmed, as they pose a continuous threat to the surrounding domestic ruminant populations.

Specifically, the invasive liver fluke *F. magna* was first documented in the CR around 1910 in fallow deer. Following this initial discovery, subsequent infections have predominantly been reported in red, fallow, and roe deer across several regions, particularly within the wetland and forest ecosystems of South Bohemia, Central Bohemia, and Plzeň including areas near the German border (24, 28, 36, 85). This invasive species has thus maintained a stable presence in the wildlife of the CR for over a century. Our findings further support the continued persistence of these endemic hotspots, especially in Southern and Central Bohemia regions.

Furthermore, our research has confirmed the presence of *F. magna* eggs in roe deer feces, thereby challenging previous assumptions regarding their classification as aberrant hosts. Historically, infections in roe deer have been associated with severe, frequently fatal consequences characterized by the absence of pseudocyst formation and egg production. However, recent evidence suggests the initiation of adaptive processes within this species (86–88). Increasing observations of pseudocyst development and egg excretion indicate a potential shift toward a more chronic form of fascioloidosis with reduced mortality rates. This adaptation could support the parasite’s long-term persistence and transmission, potentially involving a broader spectrum of vertebrate hosts in sustaining and disseminating this invasive trematode over time.

Although comprehensive data on the occurrence of *F. hepatica* in wild ruminants in the CR are lacking, the absence of this parasite in our samples is surprising, especially considering its common occurrence in many parts of Europe (44, 45, 89, 90). However, a recent study conducted in Slovakia, a neighboring country to the CR, examined 782 fecal samples and did not confirm the presence of this parasite in wild ruminants as well (48).

Historically regarded as uncommon in temperate climates, *C. daubneyi* has been increasingly documented in domestic and wild ruminants, indicating an expanding geographical range and potential implications for European ecosystems (29–31). In our study, this trematode was identified in two instances—specifically, in a mouflon and a fallow deer, yielding an overall prevalence of 0.2%. These findings corroborate research conducted in Ireland, which reported the presence of *C. daubneyi* in various deer species, including fallow deer, suggesting that these animals may serve as reservoirs for this trematode (35). Furthermore, our findings are consistent with a recent study conducted in the CR by Rehbein et al. (36), which reported *C. daubneyi* in 4 of 471 red deer samples and 1 of 1 sika deer sample (total prevalence = 0.8%), compared to the prevalence of 21.5% in cattle (53 of 247 samples) in Šumava National Park and surrounding pastures, located near the German border. This significant disparity further suggests a potential pathogen spillover from livestock to wildlife, underscoring the interconnectedness of wildlife and livestock populations in the transmission dynamics of parasites.

The last trematode species included in our study was *D. dendriticum*, which was selected to validate the specificity of the detection systems employed for liver flukes. Data on the distribution and prevalence of this fluke in the CR are limited, though it is known to commonly infect mouflon, as seen in a Slovak study showing a 30.83% infection rate (48). Our study corroborated this finding, recording a significantly lower prevalence rate of 6.4%. Conversely, roe deer and red deer showed minimal or no infection, aligning with a study by Iglódyová et al. (48) but contrasting with findings from Romania (49). Notably, fallow deer in our study had a 5.4% prevalence, absent in other referenced studies.

An interesting finding was the detection of co-infections of *A. sidemi* and *Haemonchus* spp., for which sufficient data are not yet available. Our study identified four cases of co-infection of these nematodes in roe deer, aligning with previous reports. Kuzmina et al. (19) documented both species co-infecting roe deer in Ukraine, while Lehrter et al. (75) reported analogous concurrent infections in France, encompassing both roe deer and red deer. Furthermore, the lower-than-expected co-infection rates between these closely related nematodes imply the presence of a competitive exclusion mechanism, wherein one species may inhibit the establishment or proliferation of the other. This observation is consistent with prevailing ecological theory, which posits that closely related species occupy more similar ecological niches than distantly related species, thereby constraining their ability to coexist due to direct resource competition (91, 92).

Regarding diagnostic methods, multiplex real-time PCR assays represent a significant step forward in parasitological research, particularly when compared to traditional techniques based on morphological identification. The molecular assays offer enhanced sensitivity and specificity, reducing subjective errors in taxonomic classification (52, 57, 58). The ability to detect multiple parasite targets (both nematodes and trematodes) within a single sample processing workflow optimizes the efficiency of large-scale, high-throughput sample screening. Additionally, these assays enable intravital analysis by directly examining fecal samples, circumventing the need for animal death.

Despite the advantages of molecular methods, several limitations must be considered. The integrity and freshness of fecal samples are critical, as DNA degradation poses a significant risk when exposed to adverse weather conditions, leading to desiccation. Such degradation can compromise the accuracy of analyses, potentially resulting in an underestimation of parasite prevalence. We have increased the number of samples and sampling locations in each region to address this issue. Additionally, we conducted collections during the colder months, as done by Novobilský et al. (85) in the territory of the CR and based on data from other studies on seasonal dynamics (71, 93).

Furthermore, methods that detect parasites in feces are inherently limited, as they primarily identify only their propagative stages (e.g., eggs, larvae, oocysts) and can be influenced by the intermittent shedding of these stages, which is contingent upon various factors, including host physiology, environmental influences, and the biology of the parasites themselves (71, 93–95). Comparative analysis with studies employing necropsy methods, such as Magdálek et al. (18), highlights this limitation. While our study identified *A. sidemi* in red deer (*p* = 30.7%), roe deer (*p* = 5.4%), and fallow deer (*p* = 7.7%), Magdálek et al. (18) reported significantly higher prevalence rates in the same ruminant species (red deer: *p* = 65.0%; roe deer: *p* = 75.0%; fallow deer: *p* = 74.2%). Although their study examined a smaller sample size, necropsy provides the most comprehensive and accurate information on parasitic load due to its ability to facilitate direct examination of organs and tissues, detect all developmental stages of parasites, and precisely quantify infection intensity.

When detecting liver flukes such as *F. magna* and *F. hepatica* and rumen flukes like *C. daubneyi*, very low egg counts per gram (EPG) are commonly observed in fecal samples of wild ruminants. For instance, available data reports an average egg count of 12 eggs per gram (EPG) for *F. magna* in the feces of red deer (85), whereas *D. dendriticum* is associated with a higher average egg count of 27 EPG (48). Although a nested PCR approach has been employed to enhance the sensitivity of detection for these flukes (excluding the *D. dendriticum* system), the possibility remains that such low egg counts may not be present and detected in the analyzed input quantity of 1 gram of feces. While our approach is appropriate for identifying affected locations in a large-scale study, future research aimed at gaining a more comprehensive understanding of the epidemiology of these parasites will necessitate the integration of multiple methodologies (e.g., sedimentation using larger quantities of input material followed by real-time PCR), despite the increased labor intensity and time requirements.

## 5 Conclusion

This comprehensive study, utilizing advanced molecular techniques, provides crucial insights into the current prevalence and spread of important helminth infections in wild ruminants in the CR, representing a unique effort at the national level. The notably high prevalence of *A. sidemi*, particularly in red deer, highlights the invasive potential of this nematode and its capacity to establish itself in new environments and among new hosts. The widespread presence of *Haemonchus* spp. across various regions of the CR and host species further underscores the pervasive nature of this infection, emphasizing the necessity for continuous surveillance to mitigate potential outbreaks that could potentially adversely affect both wild and domestic ruminant health, especially in the context of evolving anthelmintic resistance. Conversely, the low detection rates of liver and rumen trematodes, such as *F. magna*, *D. dendriticum*, and *C. daubneyi*, suggest a more localized distribution influenced by specific ecological and environmental factors. Despite their lower prevalence, the persistent occurrence of these trematodes poses an ongoing risk in locations where wildlife and livestock are sympatric. Overall, our study offers essential data for mitigating the impacts of helminth infections, particularly in the context of increasing biological invasions.

## Data Availability

The original contributions presented in the study are included in the article/Supplementary material, further inquiries can be directed to the corresponding author.

## Footnotes

^1^GenBank: NCBI Sequence Database (National Center for Biotechnology Information, Bethesda, MD, USA). Available from: https://www.ncbi.nlm.nih.gov/genbank/ [Accessed December 11, 2024].

^2^OligoAnalyzer 3.1 (Integrated DNA Technologies, Coralville, IA, USA). Available from: https://www.idtdna.com/ [Accessed December 11, 2024].

^3^Basic Local Alignment Search Tool (National Center for Biotechnology Information, Bethesda, MD, USA). Available from: http://blast.ncbi.nlm.nih.gov/ [Accessed December 11, 2024].

## Supplementary material

SUPPLEMENTARY FILE 1 Detailed information on collected fecal samples.

SUPPLEMENTARY FILE 2 Comprehensive overview of the sequences of probes and primers used in the various detection systems, including amplicon size and specific target details.
