# Supplementary material for: Molecular uncovering of important helminth species in wild ruminants in the Czech Republic
Source: Front Vet Sci. 2025 Feb 4;12:1544270. doi: 10.3389/fvets.2025.1544270 (PMC11832707; doi:10.3389/fvets.2025.1544270)
Supplement: SUPPLEMENTARY FILE 2 — Comprehensive overview of the sequences of probes and primers used in the various detection systems, including amplicon size and specific target details. [file Supplementary_file_2.docx]

**Supplementary table 1.** Primers and probes used in the nested real-time PCR assay to detect *Fasciola hepatica* and rumen flukes.

| **Parasite Species** | **Targeted Gene** | **GenBank**   **Acc. No.** | **Position in**  **Target Gene** | **Primer/Probe Designation** | **Oligonucleotide Sequence**  **(5' to 3')** | **Product Size** | **Source/**  **Reference** |
| --- | --- | --- | --- | --- | --- | --- | --- |
| All included | *5,8S*-*ITS2*-*28S* | OQ102036.1  KU365321.1 MT423007.1 | 29 to 425 bp  47 to 444 bp  56 to 531 bp | GA1 | AGAACATCGACATCTTGAAC | 398 bp  399 bp  477 bp | (66, 67) |
|  |  |  |  | BD2 | TATGCTTAAATTCAGCGGGT |  |  |
|  | *ITS2*-*28S* | OQ102036.1 | 298 to 408 bp | Cd_ITS2_Fw | TGCTGTTGTGCCTTTGAAT | 115 bp | This study |
| *Calicophoron daubneyi* |  |  |  | Cd_ITS2_Rev | tcctGGTATTCACGTCTGAT |  |  |
|  |  |  |  | Cd_ITS2_Probe | 6FAM-TGGTGTTCTGTTACCTGACCTCGG-BHQ1 |  |  |
| Paramphisto-midae | *ITS2*-*28S* | KU365321.1 | 243 to 427 bp | Pc_ITS2_Fw* | CGTCTTGCTGGTAGCG | 189 bp | This study |
| *Fasciola hepatica* | *ITS2* | MT423007.1 | 241 to 497 bp | Fh_ITS2_Fw | GGATGCACCCTTGTCT | 257 bp | This study |
|  |  |  |  | Fh_ITS2_Rev | CGAGGTCAGGAAGACAGAC |  |  |
|  |  |  |  | Fh_ITS2_Probe | ROX-TGGTACTCAGTTGTCAGTGTGTTTGGC-BHQ2 |  |  |
| Internal amplification control | Synthetic sequence | | | IAC_Fw | AACCCCTAAACCGGATGATA |  | (69) |
|  |  |  |  | IAC_Rev | GTTTAGAATGTTTTCTCCCGTAC |  |  |
|  |  |  |  | IAC_Probe | Cy5-CTCACCTCCCCGCCCAATACTG-BHQ3 |  |  |

*For screening purposes, an additional primer, Pc_ITS2_Fw, was incorporated into the assay to function in conjunction with Cd_ITS2_Rev and Cd_ITS2_Probe. This primer was designed to non-specifically detect other representatives of the family *Paramphistomidae*, with a particular emphasis on the detection of *P. cervi* and *P. leydeni*.

**Supplementary table 2.** Primers and probes used in the nested real-time PCR assay to detect *Fascioloides magna* and *Dicrocoelium dendriticum*.

| **Parasite Species** | **Targeted Gene** | **GenBank**  **Acc. No.** | **Position in**  **Target Gene** | **Primer/Probe Designation** | **Oligonucleotide Sequence**  **(5' to 3')** | **Product Size** | **Source/**  **Reference** |
| --- | --- | --- | --- | --- | --- | --- | --- |
| *Fascioloides magna* | *5,8S*-*ITS2*-*28S* | EF534992.1 | 7 to 529 bp | Nested_ITS2_Fw | TCGGCTCGTGTGTCGATGAAGAGC | 523 bp | This study |
|  |  |  |  | Nested_ITS2_Rev | GGTAATCACGTCTGAACCGAGGTCAGGA |  |  |
|  | *ITS2*-*28S* | EF534992.1 | 323 to 460 bp | Fm_ITS2_Fw | GGTTGGTAACCAGTTATCGT | 138 bp | This study |
|  |  |  |  | Fm_ITS2_Rev | TCAGATACATGACCAAGCCT |  |  |
|  |  |  |  | Fm_ITS2_Probe | HEX-CGTTCCACTACTGTCGCTTTATCGTCG-BHQ1 |  |  |
| *Dicrocoelium*  *dendriticum* | *ITS1* | KF734772.1 | 238 to 343 bp | Dd_ITS1_Fw | GGTGCCTACCTGTCTGAT | 106 bp | This study |
|  |  |  |  | Dd_ITS1_Rev | CTCATTGAGCCTAGTACCGT |  |  |
|  |  |  |  | Dd_ITS1_Probe | 6FAM-CCTGCCTGCCCCTGTTGTG-BHQ1 |  |  |
| Internal amplification control | Synthetic sequence | | | IAC_Fw | AACCCCTAAACCGGATGATA | 137 bp | (69) |
|  |  |  |  | IAC_Rev | GTTTAGAATGTTTTCTCCCGTAC |  |  |
|  |  |  |  | IAC_Probe | Cy5-CTCACCTCCCCGCCCAATACTG-BHQ3 |  |  |

**Supplementary table 3.** Primers and probes used in the real-time PCR assay to detect *Ashworthius sidemi* and *Haemonchus* spp.

| **Parasite Species** | **Targeted Gene** | **GenBank**   **Acc. No.** | **Position in**  **Target Gene** | **Primer/Probe Designation** | **Oligonucleotide Sequence**  **(5' to 3')** | **Product Size** | **Source/**  **Reference** |
| --- | --- | --- | --- | --- | --- | --- | --- |
| *Haemonchus* spp. | *ITS2* | HQ389229.1 | 653 to 796 bp | Hc_ITS2_Fw | CGTGATGTTATGAAATTGTAAC | 143 bp | (58) |
|  |  |  |  | Hc_ITS2_Rev | CTCAGGTTGCATTATACAAAT |  |  |
|  |  |  |  | Hc_ITS2_Probe | HEX-TGCCACTATTTGAGTGTACTCAGCG-BHQ1 |  |  |
| *Ashworthius sidemi* | *ITS1* | EF467325.1 | 431 to 597 bp | As_ITS1_Fw | TCGATAAATGTGACACAAACTTT | 167 bp | (58) |
|  |  |  |  | As_ITS1_Rev | GTACGGGATATAATACTTAGTGAAGTA |  |  |
|  |  |  |  | As_ITS1_Probe | 6FAM-TGGCGTCATTGAACATGATCATTAAGGT-BHQ1 |  |  |
| Internal amplification control | Synthetic sequence | | | IAC_Fw | AACCCCTAAACCGGATGATA | 137 bp | (69) |
|  |  |  |  | IAC_Rev | GTTTAGAATGTTTTCTCCCGTAC |  |  |
|  |  |  |  | IAC_Probe | Cy5-CTCACCTCCCCGCCCAATACTG-BHQ3 |  |  |

All primers and probes utilized in this study were purified using high-performance liquid chromatography (HPLC) and were obtained from Sigma-Aldrich (St. Louis, MO, USA).
